# Pre-reproductive maternal enrichment influences offspring developmental trajectories: motor behavior and neurotrophin expression

**DOI:** 10.3389/fnbeh.2014.00195

**Published:** 2014-05-30

**Authors:** Paola Caporali, Debora Cutuli, Francesca Gelfo, Daniela Laricchiuta, Francesca Foti, Paola De Bartolo, Laura Mancini, Francesco Angelucci, Laura Petrosini

**Affiliations:** ^1^Department of Psychology, University “Sapienza” of RomeRome, Italy; ^2^I.R.C.C.S. Santa Lucia FoundationRome, Italy; ^3^Department of Systemic Medicine, University of Rome “Tor Vergata”Rome, Italy

**Keywords:** environmental enrichment, maternal experiences, motor development, BDNF, NGF, pups, rats

## Abstract

Environmental enrichment is usually applied immediately after weaning or in adulthood, with strong effects on CNS anatomy and behavior. To examine the hypothesis that a pre-reproductive environmental enrichment of females could affect the motor development of their offspring, female rats were reared in an enriched environment from weaning to sexual maturity, while other female rats used as controls were reared under standard conditions. Following mating with standard-reared males, all females were housed individually. To evaluate the eventual transgenerational influence of positive pre-reproductive maternal experiences, postural and motor development of male pups was analyzed from birth to weaning. Moreover, expression of Brain Derived Neurotrophic Factor and Nerve Growth Factor in different brain regions was evaluated at birth and weaning. Pre-reproductive environmental enrichment of females affected the offspring motor development, as indicated by the earlier acquisition of complex motor abilities displayed by the pups of enriched females. The earlier acquisition of motor abilities was associated with enhanced neurotrophin levels in striatum and cerebellum. In conclusion, maternal positive experiences were transgenerationally transmitted, and influenced offspring phenotype at both behavioral and biochemical levels.

## Introduction

The development of mammalian brain is subjected to complex genetic and environmental influences. Specific environmental effects result from motor, cognitive, emotional, and social stimuli the individuals encounter. Enhancement of environmental stimulations, physical exercise and social interactions as occurs through the exposure to an enriched environment (EE) causes dramatic brain changes. Functional (enhanced motor and cognitive abilities, modified stress reactivity), macro-structural (increased brain weight and cortical thickness), micro-structural (increased dendritic arborisation, spine number, synaptic density, and neurogenesis), and molecular (changes in gene expression, modulation of neurotrophic factors and neurotransmitter systems) modifications have been described following EE exposure (Nithianantharajah and Hannan, 2006; Petrosini et al., 2009; Baroncelli et al., 2010; Simpson and Kelly, 2011).

At odds with the abundant literature demonstrating the beneficial effects of environmental enrichment applied immediately after weaning or even in adulthood, the transgenerational proactive effects have been scarcely investigated (Dell and Rose, 1987; Arai et al., 2009; Leshem and Schulkin, 2012; Mashoodh et al., 2012; Mychasiuk et al., 2012). The transfer of phenotypic traits acquired by parents and transmitted to offspring is a debated process in biology since its promotion by Lamarck (1809). The epigenetic phenomenon that imprints parental environmental experiences on the genome and leads to a modified phenotype that can persist over many generations, may be analyzed by taking into account biochemical and behavioral modifications of the offspring (Weaver, 2007).

Among all molecules involved in brain development in normal or complex environment, neurotrophins, as the Brain Derived Neurotrophic Factor (BDNF) and the Nerve Growth Factor (NGF), influence brain development, functional plasticity (synapse formation, axonal outgrowth, and circuital remodeling), as well as plastic mechanisms involved in learning and memory, and in responses to stress or injury (Dreyfus, 1998; McAllister et al., 1999; Sofroniew et al., 2001; Segal, 2003; Blum and Konnerth, 2005). The expression of offspring neurotrophins is modified by early experiences, either negative (i.e., maternal maltreatment) or positive (i.e., communal nesting) (Roth et al., 2009; Branchi et al., 2011).

With regard to behavioral modification, parental manipulations either negative, as food restriction, benzodioxin exposure or maternal stress, or positive, as environmental enrichment, alter the maturation of reflexes and motor coordination as well as the cognitive and emotional offspring development (Patin et al., 2004; Nishijo et al., 2007; Zhang et al., 2010; Mychasiuk et al., 2011, 2012; Qin et al., 2011). Namely, several reports describe the effects of prenatal EE exposure, using different rat strains, schedules and protocols of environmental enrichment, and various behavioral tests (McKim and Thompson, 1975; Kiyono et al., 1985; Koo et al., 2003; Mychasiuk et al., 2012; Rosenfeld and Weller, 2012). All of these reports show that the exposure of the pregnant mother to an EE has the potential to prepare the fetus to cope with a specific environment. Similar results have been found combining pre- and post-natal environmental enrichment (Welberg et al., 2006; Leshem and Schulkin, 2012). In these conditions the environmental experiences influence not only the mother but also the fetus firstly, and the pup later. Therefore, it is hard to distinguish pre- from post-natal effects, indirect from direct maternal effects on the offspring (Welberg et al., 2006). The enhanced environmental experiences the mother is exposed to may exert their influence in a multifaceted way. Firstly, the fetus has a restricted, yet existent, ability to directly perceive environmental messages through sounds, taste, smell or mechanic stimulations (Alberts and Ronca, 1993; Youngentob and Glendinning, 2009; Kim et al., 2013). Moreover, the fetus is indirectly affected by the environmentally-induced changes in mother's behavior, physiology (nutrition, HPA axis), and mood (Van den Bergh et al., 2005; Weinstock, 2005; Brummelte and Galea, 2010; Stachowiak et al., 2013; Rathod et al., 2014). Lastly, pup's post-natal development is affected by the maternal care that in turn is sculptured by the pre-natal or post-natal environment the mother is exposed to (Francis and Meaney, 1999; Meaney, 2001; Francis et al., 2002; Macrì and Wurbel, 2006; Sparling et al., 2010; Rosenfeld and Weller, 2012).

On such a basis, it seemed interesting to investigate whether a maternal EE exposure limited to the pre-reproductive period may affect offspring phenotype, thus avoiding any direct effect of EE exposure either on the fetus (pre-natal exposure) and on the pup (post-natal exposure). To this aim, female rats were reared in an EE from weaning to sexual maturity, then housed in standard condition from mating with standard-reared males onwards. In offspring, postural, locomotor and behavioral maturation was analyzed from the first postnatal day (pnd 1) to the weaning (pnd 25). Examining the maturation of motor behaviors has allowed a proper assessment of the influence of pre-reproductive maternal enrichment on the developmental motor sequences of offspring (Flagel et al., 2002; Heyser, 2004; de Souza et al., 2004; Ellenbroek et al., 2005; So et al., 2010). A variety of motor functions was examined to verify whether the various aspects of motor behaviors were influenced to a similar extent by the maternal rearing condition. Furthermore, given the critical role of neurotrophins in survival, maintenance, and maturation of neurons (Lindsay et al., 1994; Lewin and Barde, 1996), the expression of BDNF and NGF was evaluated in the cerebral cortex, hippocampus, striatum and cerebellum, brain areas involved in motor and behavioral development and sensitive to environmental challenges, such as the exposure to an EE (Pham et al., 1999, 2002; Angelucci et al., 2009).

## Materials and methods

### Maternal housing conditions

**Figure 1:**
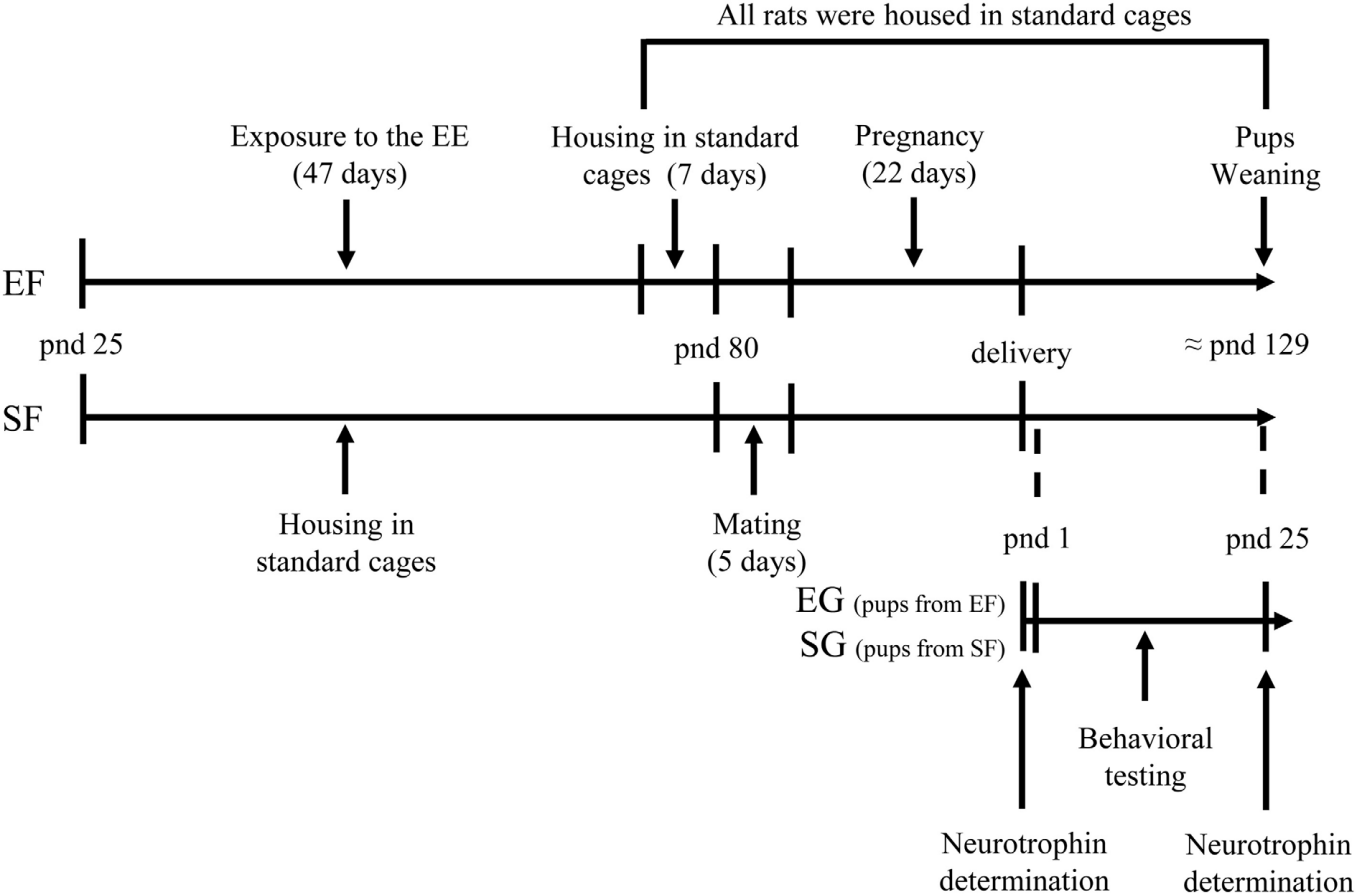
**Timeline of the experimental procedure**. Experimental groups of female rats according to different pre-reproductive rearing conditions (EF, enriched females; SF, standard females. Duration: ≈ 7 weeks). Experimental groups of male pups (EG, enriched group; SG, standard group), behavioral testing (postural, locomotor, and behavioral assessment), and biochemical analyses (neurotrophin determination at birth and at weaning).

Global timing of experimental procedures is reported in Figure 1.

At weaning, female Wistar rats were randomly assigned to enriched or standard rearing conditions.

From pnd 25 to pnd 72, the Enriched Females (EF) were reared in groups of ten in a large cage (100 × 50 × 80 cm) with an extra level constructed of galvanized wire mesh and connected by ramps to create two interconnected levels, following the enrichment conditions previously described (Petrosini et al., 2009; Cutuli et al., 2011; Foti et al., 2011). The cage contained wood shavings, a running wheel, a shelter (a house-shaped toy with a concave opening in which the rat could enter), colored plastic toys (red or green small balls, little bells, jingle noise-maker playthings, and ropes), and small objects (transparent rat igloo, colored bricks, cubes, tunnels, a mirror, and two platforms). Throughout the enrichment period, the shelter and running wheel were kept stable in the cage, whereas the toys and constructions were changed twice a week. Once a week, the feeding boxes and water bottles were moved to different cage areas to encourage explorative behaviors. Furthermore, each enriched animal was handled daily for at least 10 min. On pnd 72, the EF were pair-housed in standard cages (40 × 26 × 18 cm) for a week to become accustomed to the standard cages before mating.

The Standard-reared Females (SF) were pair-housed in a standard cage containing wood shavings and a red plastic tube. Feeding boxes and water bottles were kept in the same position. SF received the usual care by the animal house staff without prolonged manipulations. This procedure avoided an impoverished rearing and also SF were accustomed to the human contact.

A 12/12 h dark/light cycle (light on between 07:00 and 19:00 h) was applied to both enriched and standard conditions. Food and water were provided *ad libitum*. All efforts were made to minimize animal suffering and reduce the number of animals that were used, per the European Directive (2010/63/EU). All procedures were approved by the Italian Ministry of Health.

Before mating all females were weighted. For mating, from pnd 80 to pnd 85 (six days), each EF and SF in oestrus stage was caged with a standard-reared male rat (≈300 g). Afterwards, the males were removed, and the females were maintained in the standard home cages (40 × 26 × 18 cm) throughout pregnancy, delivery and until offspring's weaning, at pnd 25.

### Experimental groups of pups

The day of delivery (pnd 0), litter size and sex ratio were noted. Then, culling was quickly performed by reducing the litters to six males and two females. The litters with less than six males were not used.

Depending on the maternal rearing conditions, two groups of pups were obtained: “enriched” group (EG), encompassing male pups of enriched females; standard group (SG), encompassing male pups of standard females. Note that the difference in rearing conditions concerned the mothers in their pre-reproductive phase and not the pups that were all reared in standard conditions.

For neurotrophin determination at pnd 0, 5 culled male pups from each group (2–3 pups/litter from two litters) were sacrificed.

For behavioral analyses, 24 male pups of each group (6 male pups/litter from 4 litters born within the same 12-h period) were used. At the end of behavioral evaluation, 5 male pups from each group were sacrificed for neurotrophin determination.

### Behavioral testing

From pnd 1 to pnd 25, EG and SG pups were separated daily from the dams one by one for a maximum of 20 min. They were tested for their postural, locomotor, and complex behaviors in a warmed environment (30–32°C) between 9 AM and 3 PM (Petrosini et al., 1990; Heyser, 2004; Sousa et al., 2006).

The instrument for behavioral assessment consisted of a battery of tests examining: physical development (body weight, eye opening, fur appearance, incisor eruption); reflex appearance (cliff avoidance, negative geotaxis, vestibular drop); development of quadrupedal stance (head and shoulder elevation) and locomotion (pivoting, crawling, and quadrupedal locomotion); development of complex motor behaviors (ascending a ladder, crossing a narrow bridge, suspension on a wire). Swimming performance (direction and limb use) were also evaluated.

To supplement direct behavioral observations, videos were recorded throughout the entire testing cycle. To avoid any eventual order effect, the testing sequence was in random order for each pup on each measure. Researchers unaware of the individual pup group assignment attributed the dominant behavior in each observation period to a specific category. Categorization was considered reliable only when judgments were consistent (inter-rate reliability > 0.9).

#### Test battery

Physical development: *Body weight* was measured daily until pnd 25. *Eye opening, fur appearance*, and *incisor eruption* were evaluated by visual inspection.

***Reflex appearance***. *Cliff avoidance*: the pup was placed on an edge with forepaws and nose just over the edge. The time to retract itself by means of backward and/or sideward movement was recorded. The max allotted time was 60 s.—*Negative geotaxis*: the rat was placed on an inclined plane (25°) with its head pointing downwards. The time to turn to face up the slope was recorded. The max allotted time was 60 s.—*Vestibular drop*: the animal was suspended by its tail and it raised its head to tail level by arching its body sideways. The time to arch its body left and right to tail level was recorded. For all these reflexes, the pnd of appearance was noted.

***Development of quadrupedal stance and locomotion.*** Each animal was placed on a board and video-recorded for 180 s to analyze quadrupedal stance (*head* and *shoulder elevation* times) and locomotion categories (*pivoting, crawling* and *quadrupedal locomotion*) by using Ethovision XT (Noldus, the Netherlands) from pnd 3 to pnd 14. *Pivoting* consisted of turning movements by broad swipes with the forepaws, body weight supported by only one hindlimb used as a pivot, and pelvis anchored to the ground. *Crawling* consisted of dragging themselves forward or pushing backward by undulating movements of the trunk, hindlimbs were often dragged in extended position with soles of the feet facing upward. *Quadrupedal locomotion* consisted of fluent and swift forward movements, with all limbs supporting the whole body and the pelvis elevated. The appearance (or disappearance) of locomotion categories was scored as the prevalent behavior according to the rating scale described in Table 1.

**Table 1:**
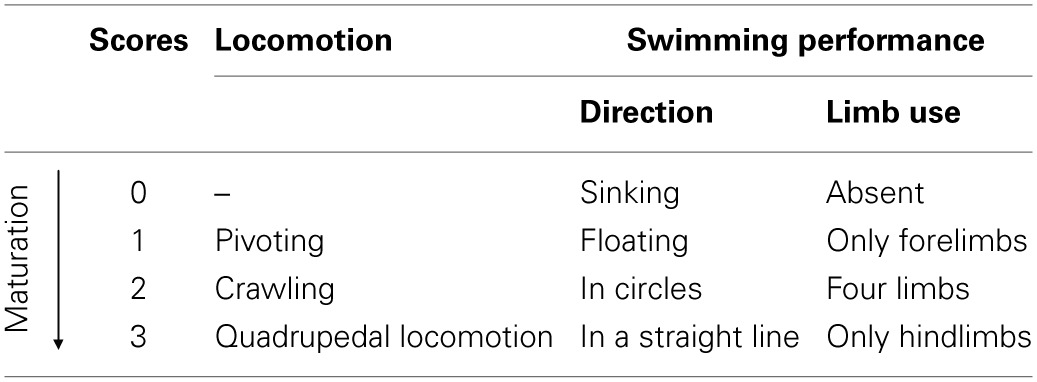
**Rating scale for locomotion and swimming performance**.

***Development of complex motor behavior***. *Ascending a ladder*: the rat was placed on a steel ladder (14 × 40 cm, with 30 rungs, 1 cm apart) at an angle of 25° with its top in contact with a platform, containing a female littermate of the tested animal. The max allotted time was 180 s. The day of the successful arrival on the platform was recorded.—*Crossing a narrow bridge*: the pup was placed on the start platform connected by a plywood bridge (60 × 1 × 3 cm) to a goal platform containing a female littermate of the tested animal. The ability to traverse the bridge within the allotted 180 s was evaluated and the day of the first successful crossing was recorded.—*Suspension on a wire*: the rat was suspended by the forepaws on a wire (3 mm thin and 100 cm long) extended horizontally between two poles (50 cm high). The max allotted time was 180 s. Suspension time was recorded.—*Swimming performance*: the rat was immersed in a glass tank (100 × 50 × 20 cm) filled with 35°C water. Water level was chosen to avoid contact with the tank bottom during swimming. The animal was gently released by hand in the water and allowed to swim freely about the tank. Two swimming parameters (*swimming direction* and *limb use*) were evaluated and scored according to the rating scale described in Table 1.

### Tissue dissection

The animals were decapitated and the brains were quickly removed and dissected on ice using a binocular dissection microscope. The following brain regions were collected according to Glowinski and Iversen's method (1966): cortex and hippocampus (pnd 0); frontal cortex, hippocampus, striatum and cerebellum (pnd 25). Striatal and cerebellar neurotrophin expression was not analyzed at pnd 0, because of the very weak BDNF and NGF expression in these extremely immature areas in the very early postnatal days, as previously described (Knüsel et al., 1994; Katoh-Semba et al., 1997; Sherrard and Bower, 2002). All brain regions were extracted in 1 ml extraction buffer/100 mg tissue. Brain tissue samples were homogenized in an ice-cold lysis buffer containing 137 mM NaCl, 20 mM Tris–HCl (pH 8.0), 1% NP40, 10% glycerol, 1 mM phenylmethanesulfonylfluoride (PMSF) 10 mg/ml aprotinin, 1 mg/ml leupetin, and 0.5 mM sodium vanadate. The tissue homogenate solutions were centrifuged at 14000 g for 25 min at 4°C. The supernatants were collected and stored at −80°C until analyses.

### BDNF and NGF determination by enzyme-linked immunosorbent assay (ELISA)

Concentrations of BDNF and NGF proteins were assessed using a two-site enzyme immunoassay kit (Promega, Madison, WI, USA). In brief, 96-well immunoplates (NUNC) were coated with 50 μ l/well with the corresponding capture antibody which binds the neurotrophin of interest, and stored overnight at 4°C. The next day serial dilutions of known amounts of BDNF and NGF ranging from 0 to 500 pg/ml were performed in duplicate to generate a standard curve. Then the plates were washed three times with wash buffer and the standard curves and supernatants of brain tissue homogenates were incubated in the coated wells (100 μ l each) for 2 h at room temperature (RT) with shaking. After additional washes, the antigen was incubated with second specific antibody for 2 h at RT (BDNF) or overnight at 4°C (NGF), as specified in the protocol. The plates were washed again with wash buffer and then incubated with an anti-IgY HRP for 1 h at RT. After another wash, the plates were incubated with a TMB/Peroxidase substrate solution for 15 min and phosphoric acid 1M (100 μ l/well) was added to the wells. The colorimetric reaction product was measured at 450 nm using a microplate reader (Dynatech MR 5000, Germany). Neurotrophin concentrations were determined from the regression line for the neurotrophin standard (ranging from 7.8 to 500 pg/ml-purified mouse BDNF or NGF) incubated under similar conditions in each assay. Cross-reactivity with other related neurotrophic factors, for example, NT-3 and NT-4 was less than 3%. Neurotrophin concentration was expressed as pg/g wet weight and all assays were performed in triplicate.

### Statistical analysis

Statistical analyses were performed by using STATISTICA 8 (StatSoft). The data expressed as mean ± s.e.m. were first tested for normality (Wilk-Shapiro's test) and homoscedasticity (Levene's test), and then analyzed by one-way or two-way ANOVAs for independent (group) and repeated (pnd) measures followed by Tukey's HSD test.

For locomotion and swimming performance, the comparisons between groups were performed by means of the Mann-Whitney *U* test.

To control for the alpha inflation the proportion of type I errors among all rejected null hypotheses, the False Discovery Rate (FDR) was set to 0.05. The FDR was estimated through the procedure described by Storey and Tibshirani (2003). The bootstrap procedure was used to estimate the π 0 parameter (Storey, 2004). In our results, the 0.05 level of significance corresponded to an FDR 0.01.

## Results

### Litter size and sex ratio

Enriched Females and SF showed similar body weights at weaning [*F*_(1, 18)_ = 1.77, *p* = 0.19. EF: *x* 51.94 ± 2.10 g; SF: *x* 57.16 ± 3.31 g], while at end of the EE exposure (pnd 72), EF weighted significantly less than SF [*F*_(1, 18)_ = 8.83, *p* < 0.01. EF: *x* 210.97 ± 6.64 g; SF: *x* 233.40 ± 3.58 g].

Pre-reproductive maternal enrichment did not affect either the litter size [*F*_(1, 18)_ = 2.45, *p* = 0.13. EF: *x* 10.6 ± 0.5 pups; SF: *x* 11.7 ± 0.5 pups] or the male/female ratio [percentage of male pups: *F*_(1, 18)_ = 0.0005, *p* = 0.98. EF: *x* 60.24 ± 1.92; SF: *x* 59.53 ± 4.07].

### Behavioral testing

#### Physical development

**Figure 2:**
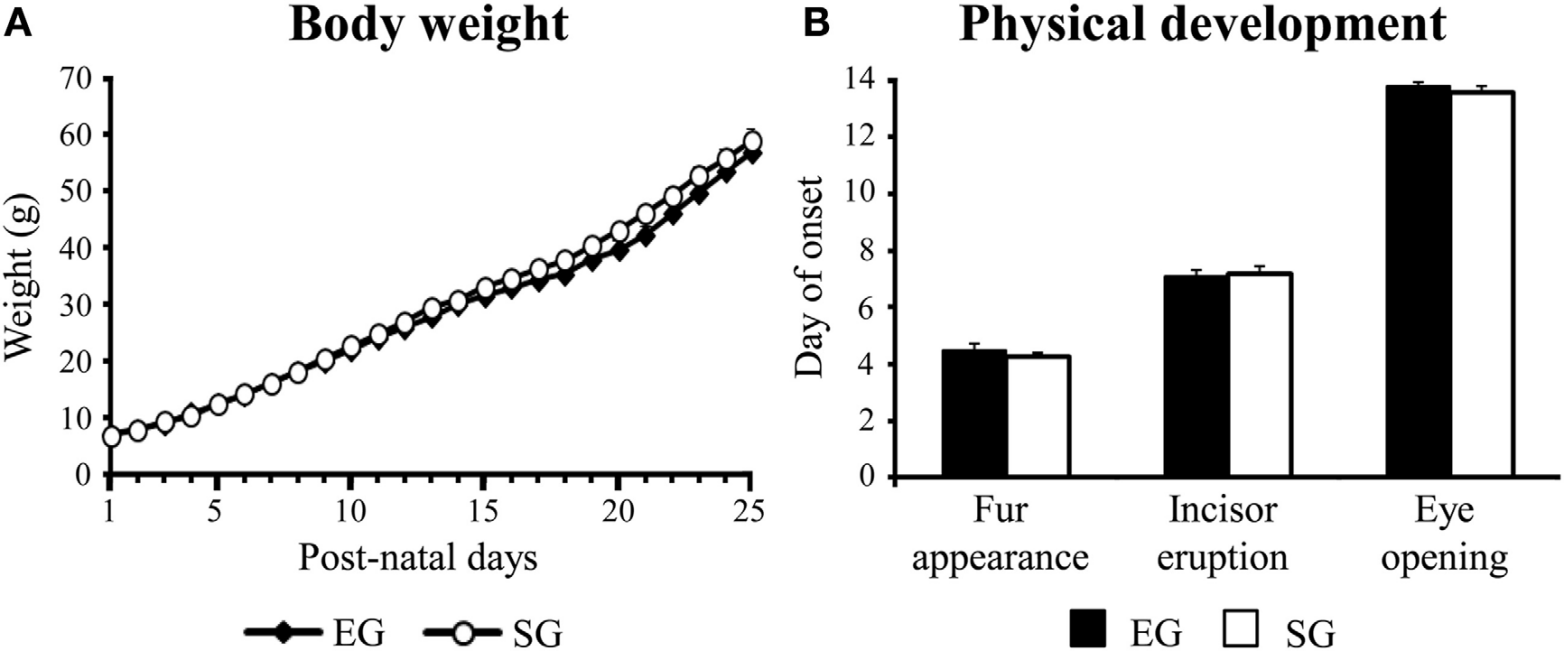
**Results of pre-reproductive maternal rearing condition on physical development**. Body weight of pups from pnd 1 to pnd 25 **(A)**; day of onset of fur appearance, incisor eruption, and eye opening **(B)**. Results are reported as mean ± s.e.m.

As indicated by a Two-Way ANOVA (group × pnd) performed on body weight of EG and SG pups, all pups increased their weight with days [*F*_(24, 1104)_ = 995.34, *p* < 0.0001], without a significant group effect [*F*_(1, 46)_ = 1.52, *p* = 0.22] and interaction [*F*_(24, 1104)_ = 0.12, *p* = 0.98] (Figure 2A).

Furthermore, EG and SG pups showed dorsal and ventral fur appearance at pnd 5 [*F*_(1, 46)_ = 2.09; *p* = 0.04], incisors at pnd 7 [*F*_(1, 46)_ = 0.05; *p* = 0.82], and opened their eyes at pnd 14 [*F*_(1, 46)_ = 1.82; *p* = 0.18], with no differences between groups (Figure 2B).

#### Reflex appearance

**Figure 3:**
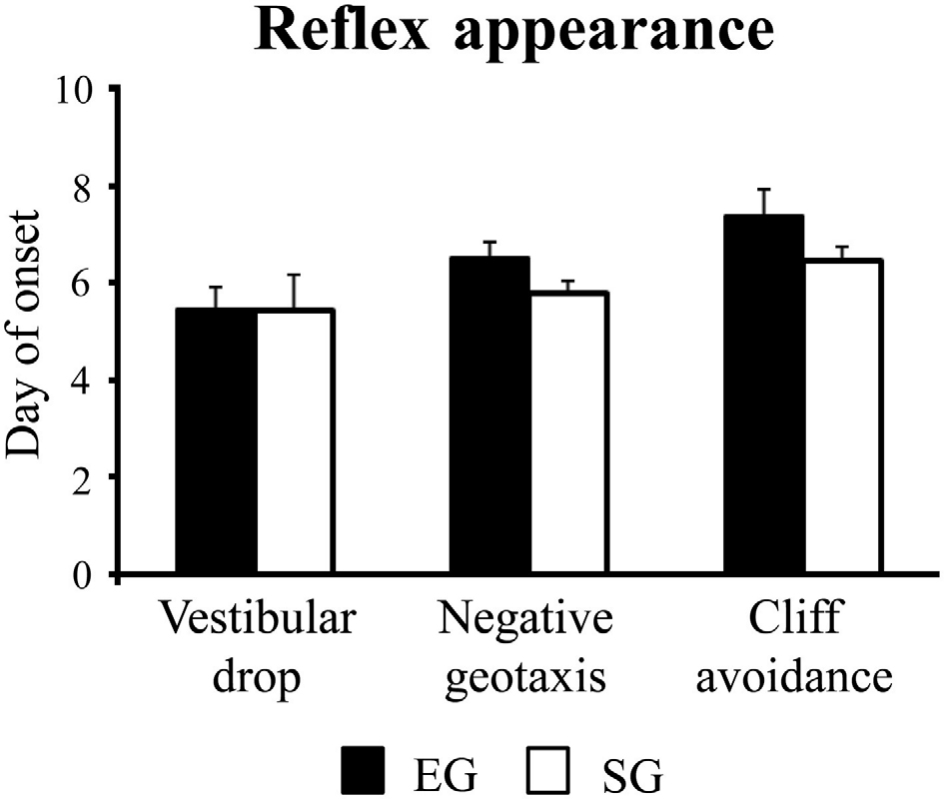
**Results of pre-reproductive maternal rearing condition on reflex appearance**. Day of onset of vestibular drop, negative geotaxis, and cliff avoidance. Results are reported as mean ± s.e.m.

Enriched group and SG pups showed a similar reflex appearance timetable, showing vestibular drop at pnd 5 [*F*_(1, 46)_ = 0.002; *p* = 0.96], negative geotaxis at pnd 6 [*F*_(1, 46)_ = 2.72; *p* = 0.11], and cliff avoidance at pnd 7 [*F*_(1, 46)_ = 2.17; *p* = 0.15] (Figure 3).

#### Development of quadrupedal stance and locomotion

**Figure 4:**
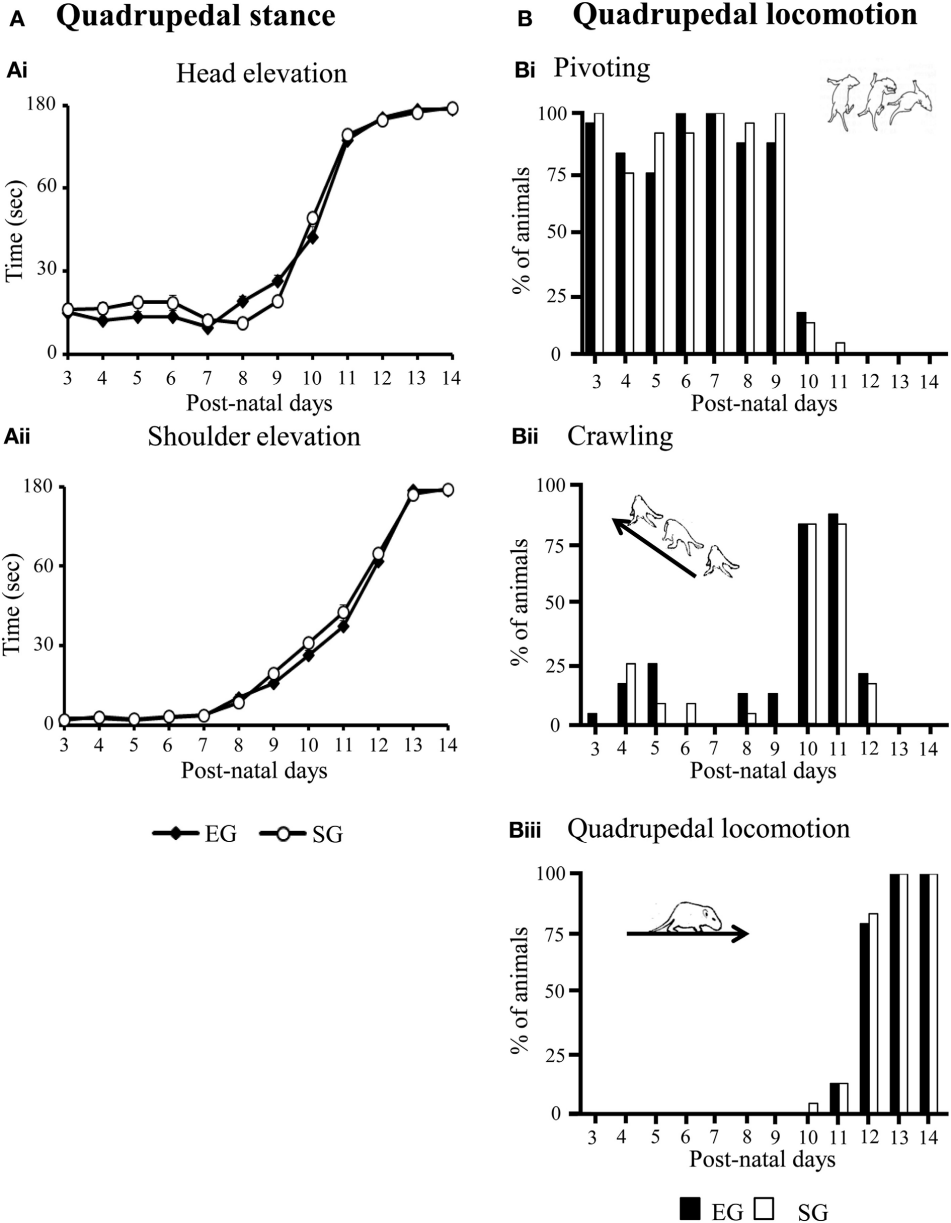
**Results of pre-reproductive maternal rearing condition on development of quadrupedal stance (A) and locomotion (B)**. Line graphs indicate elevation time of head **(Ai)** and shoulders **(Aii)**. Results are reported as mean ± s.e.m. Histograms show percentages of EG and SG pups engaged in pivoting **(Bi)**, crawling **(Bii)** or quadrupedal locomotion **(Biii)** from pnd 3 to pnd 14. Results are reported as percentages of animals displaying the behavior.

With days, EG and SG pups similarly increased head and shoulder elevation times, as indicated by Two-Way ANOVAs on elevation time (group × pnd) [Head elevation time: group effect *F*_(1, 46)_ = 0.77, *p* = 0.39; pnd effect *F*_(11, 506)_ = 705.59, *p* < 0.00001; interaction *F*_(11, 506)_ = 1.09, *p* = 0.16. Shoulder elevation time: group effect *F*_(1, 46)_ = 2.85, *p* = 0.10; pnd effect *F*_(11, 506)_ = 1508.42, *p* < 0.00001; interaction *F*_(11, 506)_ = 1.94, *p* = 0.03] (Figures 4Ai, ii).

No significant difference between groups was found in the development of quadrupedal locomotion. Namely, at pnd 3–9, most EG and SG pups turned with circular motions (*pivoting*), at pnd 10–11, they moved themselves forward or pushed backward (*crawling*), at pnd 12, all pups showed fluent and swift forward movement (*quadrupedal locomotion*) (Non-significant results of the Mann-Whitney *U* test are reported in Supplementary Table 1) (Figures 4Bi–iii).

#### Development of complex motor behaviors

**Figure 5:**
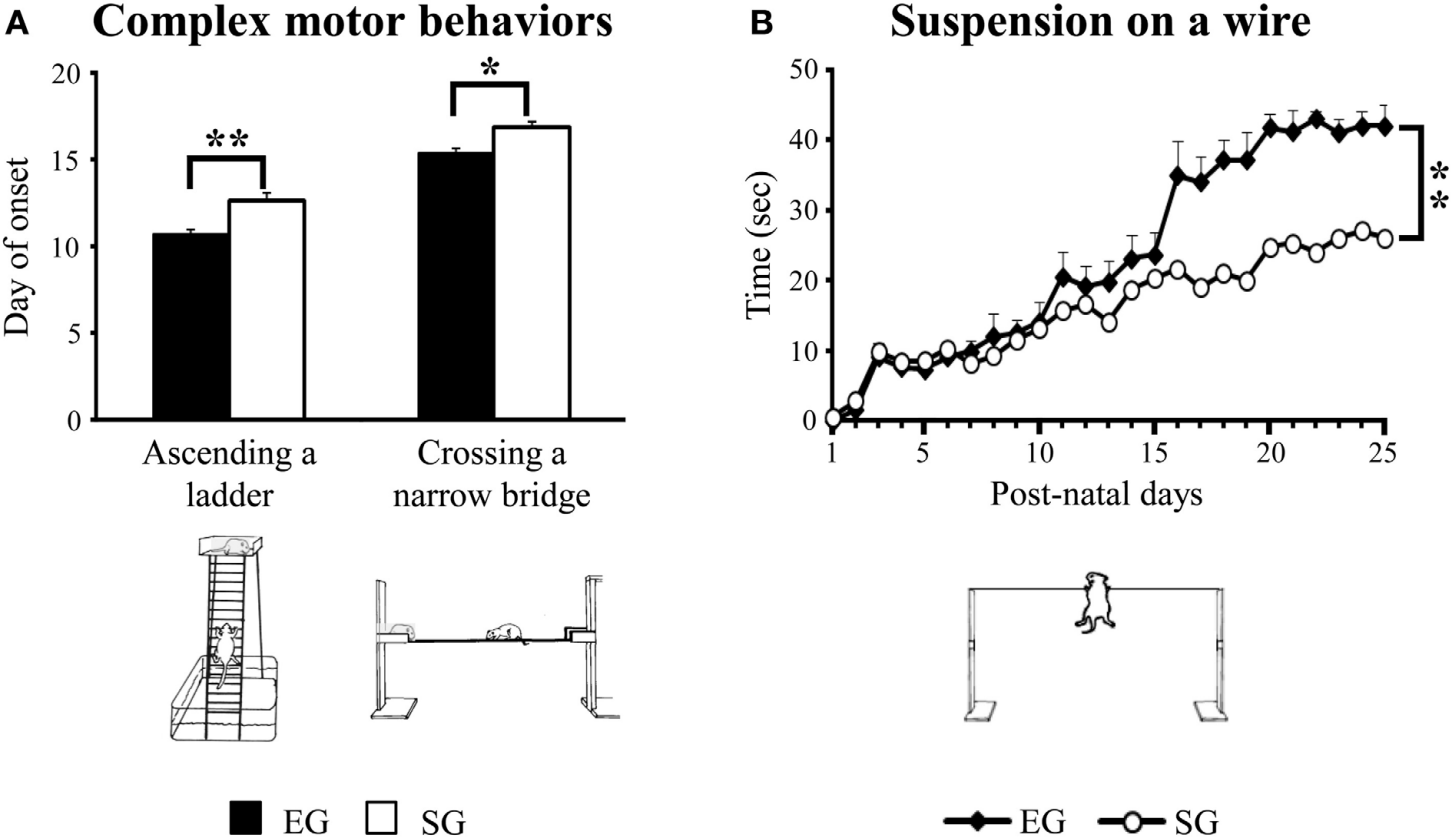
**Results of pre-reproductive maternal rearing condition on acquisition of complex motor abilities**. Day of onset of ascending a ladder and crossing a narrow bridge **(A)**; suspension time on a wire **(B)** (^*^p < 0.01; ^**^p < 0.001). Results are reported as mean ± s.e.m.

Interestingly, EG pups acquired complex motor abilities two day earlier than SG pups did. Namely, EG pups completely ascended the ladder at pnd 11 [*F*_(1, 46)_ = 14.60; *p* < 0.001] and crossed the narrow bridge from pnd 15 [*F*_(1, 46)_ = 10.77; *p* < 0.01], while SG pups acquired these behaviors at pnd 13 and pnd 17, respectively (Figure 5A).

When suspended on a wire, EG pups hung on longer than SG pups [*F*_(1, 46)_ = 9.49, *p* < 0.001]. Moreover, with days EG and SG pups increased the suspension time [*F*_(24, 1104)_ = 12.89, *p* < 0.00001]. The interaction was not significant [*F*_(24, 1104)_ = 1.66, *p* = 0.02] (Figure 5B).

**Figure 6:**
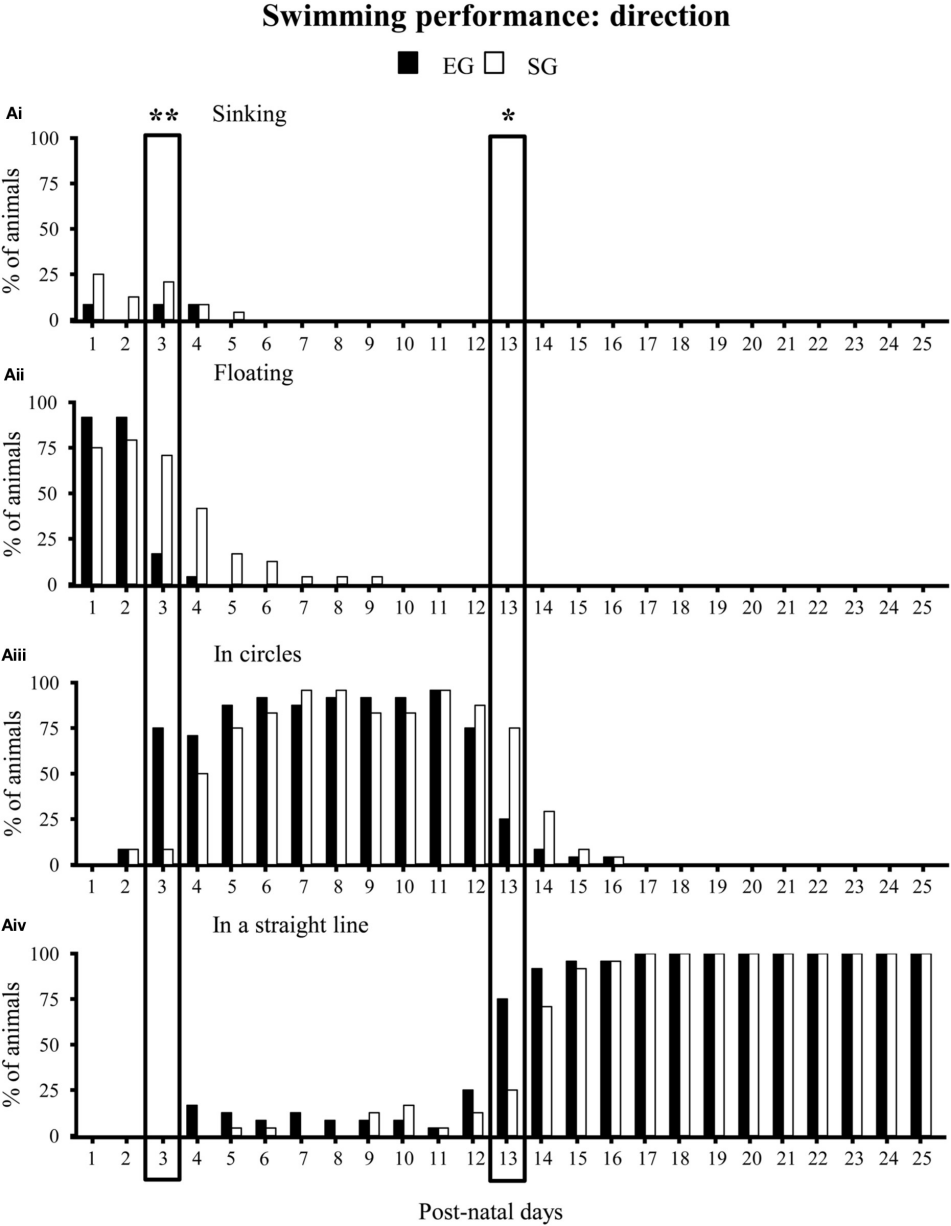
**Results of pre-reproductive maternal rearing condition on swimming direction**. Histograms show percentages of EG (*N* = 24) and SG (*N* = 24) sinking **(Ai)**, floating **(Aii)**, swimming in circles **(Aiii)** or in a straight line **(Aiv)** from pnd 1 to pnd 25 (^*^p < 0.01; ^**^p < 0.001). The two boxes indicate that at pnd 3 and pnd 13 the score distribution relative to the different swimming categories were significantly different between groups. Namely, at pnd 3 most EG pups swam in circles, while most SG pups still floated. At pnd 13, most EG pups swam in a straight line, while most SG pups still swam in circles. Results are reported as percentage of animals displaying the behavior.

**Figure 7:**
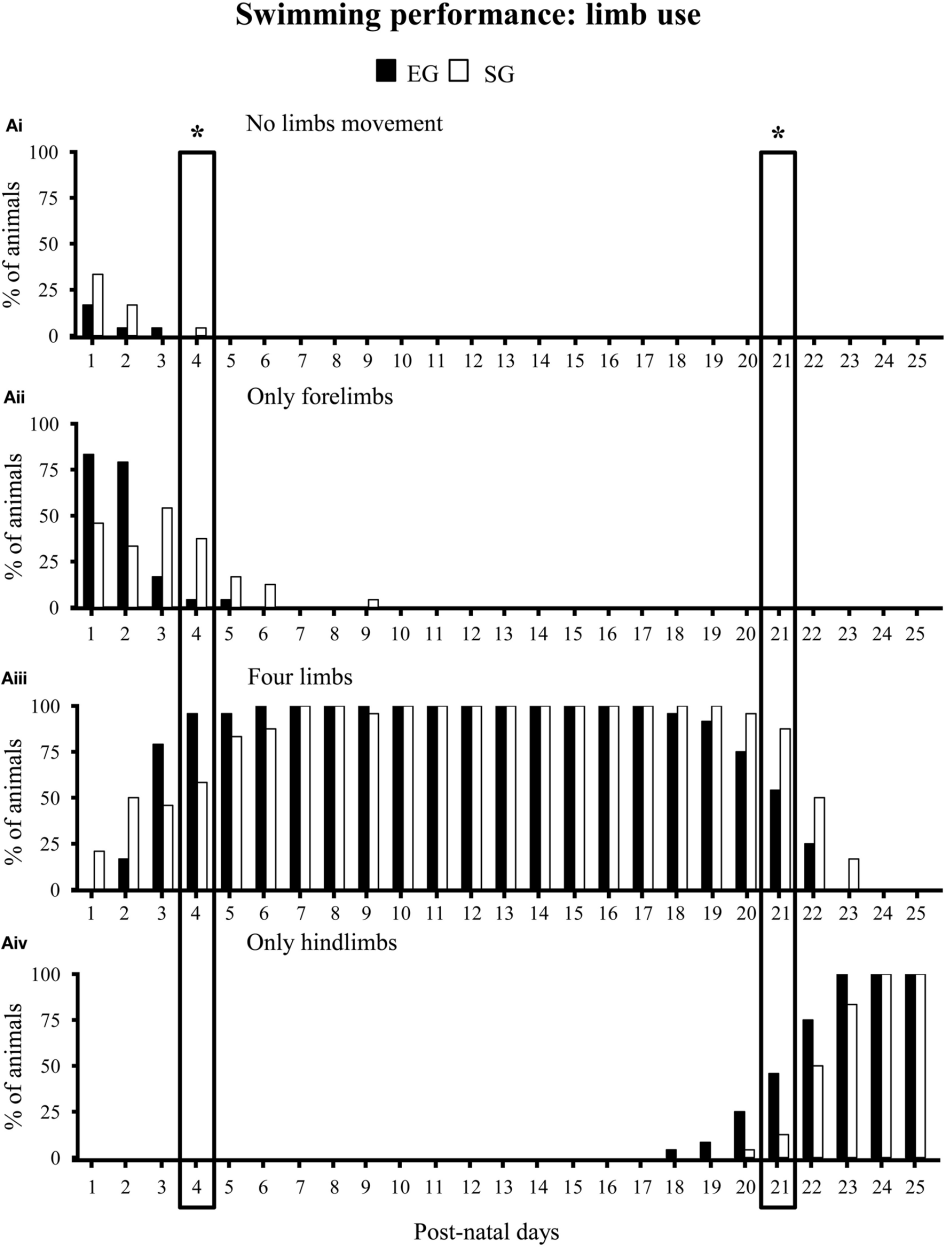
**Results of pre-reproductive maternal rearing condition on limb use during swimming**. Histograms show percentages of EG (*N* = 24) and SG (*N* = 24) pups not paddling **(Ai)**, paddling with only forelimbs **(Aii)**, four limbs **(Aiii)** or only hindlimbs **(Aiv)** from pnd 1 to pnd 25 (^*^p < 0.01). The two boxes indicate that at pnd 4 and pnd 21 the score distribution relative to the different swimming categories were significantly different between groups. Namely, at pnd 4 most EG pups swam paddling all limbs, while most SG pups still swam paddling only forelimbs. At pnd 21, most EG pups swam paddling only hindlimbs, while most SG pups still swam paddling all limbs. Results are reported as percentage of animals displaying the behavior.

With regard to swimming performance, EG pups showed earlier onset of the more adult swimming pattern in comparison to SG pups. Namely, during the very first days of observation, most EG and SG pups floated, with asynchronous limb movement that results in very little forward motion (*floating*). At pnd 3 most EG pups already swam in circles, while most SG pups still floated (Mann-Whitney *U* test: *z* = 4.16, *p* < 0.001). At pnd 13, most EG pups swam in a straight line, while most SG pups still swam in circles (*z* = 3.43, *p* < 0.01) (Figure 6). Moreover, at pnd 4 most EG pups swam paddling with all four limbs in a coordinated fashion, while most SG pups still used only the forelimbs (*z* = 3.22, *p* < 0.01). At pnd 21 most EG pups already swam moving only the hindlimbs, holding the forelimbs stationary (adult pattern of swimming), while most SG still exhibited swimming movements with all four limbs (*z* = 3.41, *p* < 0.01) (Non-significant results of the Mann-Whitney *U* test are reported in Supplementary Table 2) (Figure 7).

### BDNF and NGF protein levels

**Figure 8:**
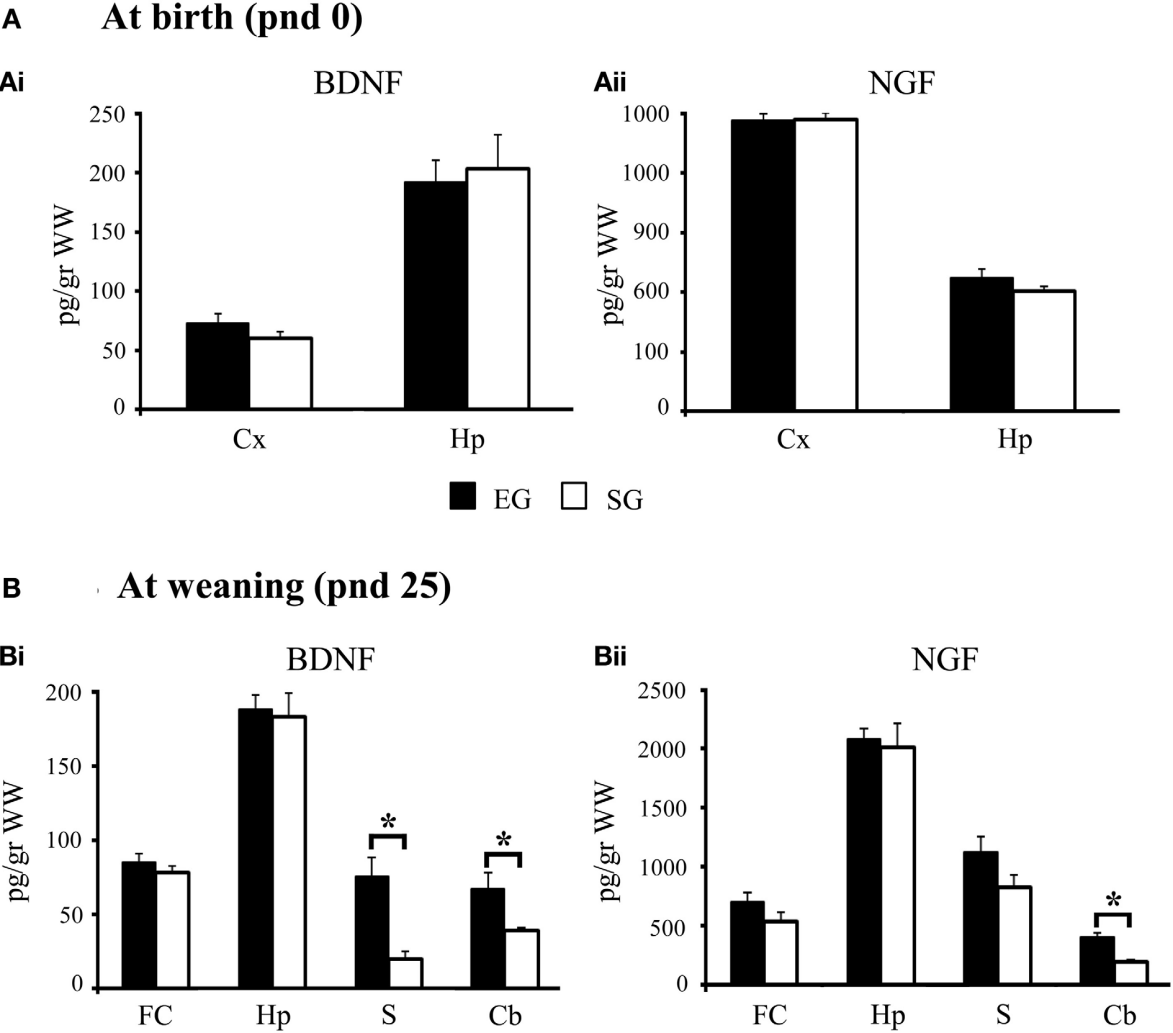
**Results of pre-reproductive maternal rearing condition on neurotrophin expression**. BDNF and NGF expression levels in cortex and hippocampus at birth **(Ai, Aii)**; BDNF and NGF expression levels in cortex, hippocampus, striatum, and cerebellum at weaning **(Bi, Bii)** (^*^p < 0.01). Results are reported as mean ± s.e.m. Cx, cortex; Hp, hippocampus; FC, frontal cortex; S, striatum; Cb, cerebellum.

At pnd 0, One-Way ANOVAs on neurotrophin levels did not show any significant difference between SG and EG pups in both cortical [BDNF: *F*_(1, 8)_ = 1.87; *p* = 0.21; NGF: *F*_(1, 8)_ = 1.82; *p* = 0.21] and hippocampal [BDNF: *F*_(1, 8)_ = 0.12; *p* = 0.74; NGF: *F*_(1, 8)_ = 0.01; *p* = 0.98] regions (Figure 8A).

At pnd 25, EG pups showed BDNF levels in the striatum [*F*_(1, 8)_ = 16.14; *p* < 0.01] and cerebellum [*F*_(1, 8)_ = 15.56; *p* < 0.01] significantly higher than SG pups. EG pups also showed higher cerebellar NGF levels [*F*_(1, 8)_ = 20.98; *p* < 0.01] than SG pups (Figure 8B). No significant differences between groups were found in BDNF levels in the frontal cortex [*F*_(1, 8)_= 0.89; *p* = 0.37] and hippocampus [*F*_(1, 8)_ = 0.09; *p* = 0.77]. Similarly, no significant differences between groups were found in NGF levels in the frontal cortex [*F*_(1, 8)_= 2.16; *p* = 0.18], hippocampus [*F*_(1, 8)_ = 0.10; *p* = 0.76], and striatum [*F*_(1, 8)_ = 3.08; *p* = 0.12] (Figure 8B).

In summary, while at pnd 0 no difference between groups was found in neurotrophin levels, at pnd 25 the cerebellar and striatal neurotrophin expression was significantly higher in EG pups.

## Discussion

**Figure 9:**
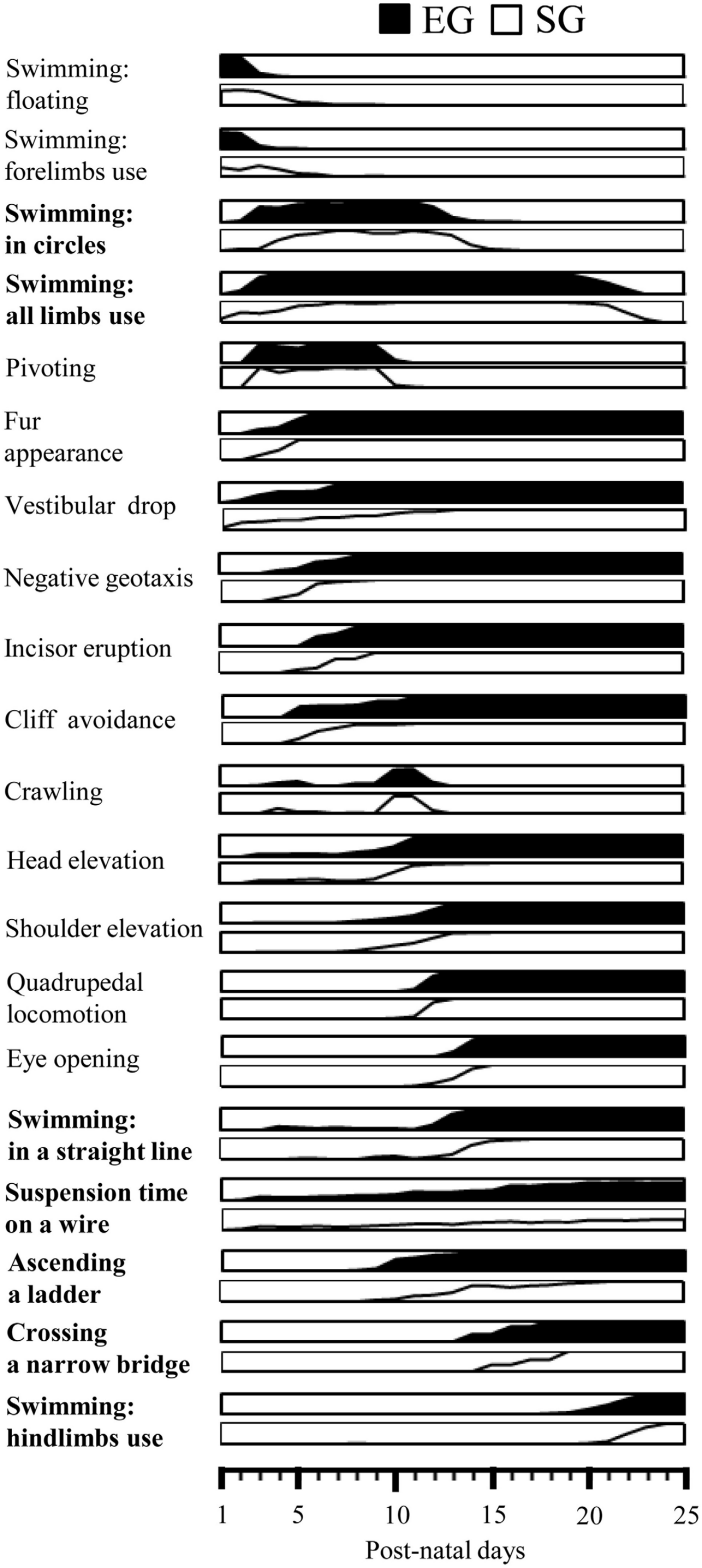
**Summary diagram of motor development of EG and SG pups**. Global timing of physical development (fur appearance, incisor eruption, eye opening), reflex appearance (vestibular drop, negative geotaxis, cliff avoidance), development of quadrupedal stance (head elevation, shoulder elevation) and locomotion (pivoting, crawling, quadrupedal locomotion) and acquisition of complex motor abilities (swimming, suspension on a wire, ascending a ladder, crossing a narrow bridge) in EG (black area) and SG (white area) pups. The behaviors significantly different between groups are indicated in bold. In each diagram the percentage of animals progressively showing the behavior are depicted.

This study aimed to evaluate whether and how positive pre-reproductive maternal experience may transgenerationally influence offspring phenotype. The maternal enrichment did influence pups' developmental trajectories (Figure 9), as indicated by their accelerated acquisition of complex motor behaviors and enhanced cerebellar and striatal neurotrophin expression at weaning. Conversely, no effect was found on cortical and hippocampal neurotrophin expression as well as on physical (fur appearance, eye opening, incisor eruption, and body weight), postural, and locomotor development. Furthermore, maternal enrichment did not influence the appearance of dynamic sensorimotor reflexes (negative geotaxis, cliff avoidance, and vestibular drop). Since these reflexes are mainly mediated by the vestibular, tactile and proprioceptive muscle systems (Altman and Sudarshan, 1975), it is possible to suggest that the maturation of these sensorimotor systems is little influenced by the pre-reproductive maternal rearing conditions.

EG and SG animals acquired quadrupedal stance and locomotion at the same rate of the maturation. The development of postural control and its intricate feed-forward adjustment of postural muscles appeared to be the basic factors for the development of quadrupedal locomotion. The rostro-caudal maturation of posture and locomotion that leads the head and then the shoulders in a raised position before the pelvis, and the forelimbs capable of coordinated movements before the hindlimbs, requires the maturation of the vestibular system, descending motor pathways, brainstem command centers, and spinal central pattern generator networks (Grillner, 2003; Clarac et al., 2004; Grillner et al., 2008). The lack of significant differences in the developmental steps of quadrupedal stance and locomotion between groups suggests that these motor behaviors and their neuronal counterparts were little affected by maternal rearing conditions.

Another type of rat “ambulation” is swimming, the development of which was accelerated by 1–2 days by the pre-reproductive maternal enrichment. Namely, EG pups swam in a circle and paddled with all four limbs already at pnd 3–4, swam in a straight line at pnd 13, and reached the adult pattern of swimming at pnd 21.

Similarly, maternal experience influenced the acquisition of other complex motor behaviors (wire suspension, ladder ascension, and bridge crossing). These abilities require proper sequencing and coordination of motor output, and exteroceptive and motivational cues. They depend on hierarchically related cortico-subcortical systems where striatum and cerebellum play crucial roles. Interestingly, the maturation of these structures lasts the first two post-natal weeks, at the end of which complex motor acts begin to be correctly executed (Gramsbergen, 2005; Grillner et al., 2005; Dehorter et al., 2012). This temporal relation evidences the cerebellar-striatal centrality in linking postural control to planning and execution of movements in order to acquire motor skills. This proposal is in line with the findings by Petrosini et al. (1990) showing that in rats neonatal cerebellar lesions performed at pnd 1 selectively affect the onset of complex motor abilities without impairing basic motor behaviors.

Interestingly, the accelerated acquisition of complex behavioral performance exhibited by EG pups was associated with increased expression of BDNF and NGF in cerebellum and striatum. However, it is hard to determine whether the biochemical differences at cerebellar and striatal level caused the differences in motor behavior or whether the different motor behavior caused the biochemical differences.

The first hypothesis is based on the possibility that the increased neurotrophin expression in cerebellum and striatum allowed developing motor skills, by promoting neuronal growth and formation of synapses and connections. Accordingly, transgenic mice over-expressing BDNF display an accelerated maturation of granule cells (Bao et al., 1999). In contrast, transgenic mice with deficient release of BDNF and neurotrophin-3 show severe impairment in cerebellar development, both at structural (survival, differentiation and migration of post-mitotic granule cells, dendritic branching of Purkinje cells) and functional (motor coordination) levels (Sadakata et al., 2007). Furthermore, in a mouse model of Huntington's disease characterized by striatal degeneration and deterioration in motor behavior, the impaired production of BDNF contributes to striatal degeneration and progressive motor abnormalities (Saudou and Humbert, 2008; Zuccato et al., 2010; Samadi et al., 2013).

On the other hand, it is not possible to neglect the opposite hypothesis on the inverse relationship between complex motor performances and neurotrophin expression in cerebellum and striatum. In fact, the early acquired complex motor abilities may have caused an increase in neurotrophin levels as a consequence of increased neuronal activity just in the structures mostly involved in the complex motor behaviors. In accordance with this hypothesis, it has been previously demonstrated that animals reared in an EE that allows the acquisition of improved complex motor behaviors show activity-dependent enhancement of BDNF and NGF levels in cerebellar and striatal areas (Angelucci et al., 2009) together with increased dendritic branching and density of spines of Purkinje cells (Lee et al., 2007) and striatal interneurons (Cutuli et al., 2011).

Unexpectedly, the pre-reproductive maternal rearing conditions did not influence neurotrophin levels in neocortical and hippocampal regions, despite the high sensitivity of these regions to parental experiences (Arai et al., 2009; Roth et al., 2009; Mychasiuk et al., 2012) and environmental enrichment (Pham et al., 1999, 2002; Angelucci et al., 2009). However, it should be considered that the lack of significant differences between groups on cortical and hippocampal neurotrophin levels did not exclude that a transgenerational effect may have occurred. In fact, mice reared in communal nesting do not display any increase in hippocampal BDNF levels when adult, even if they exhibit high levels of histone acetylation at the BDNF gene, a process known to turn on genes and increase their expression (Branchi et al., 2011). Moreover, the similar cortical and hippocampal neurotrophin levels shown by both groups could be related to the prolonged handling of the pups required by the experimental protocol. In fact, to evaluate the motor development the pups were separated from their mothers and handled for about twenty min/day, resembling an “early handling” procedure, featured by brief maternal separations (about 15–30 min), tactile and proprioceptive stimulation of pups by the experimenter, and exposure to a novel environment (Hsu et al., 2003; Daskalakis et al., 2009; Plescia et al., 2013). Notably, neurotrophin expression is markedly altered by early handling paradigms (Garoflos et al., 2005, 2007). Thus, one possibility is that the handling occurring during the evaluation of motor development could have increased the neurotrophin expression in prefrontal cortex and hippocampus of both experimental groups, creating a sort of ceiling effect restricting evidence of transgenerational effects.

Knowing the mechanisms by which environment-gene interplay is achieved allows clarifying the dynamic nature of gene regulation and biological link between experiences of an organism and individual differences in neurodevelopment and behavior (Franklin and Mansuy, 2010). The transgenerational inheritance of qualities acquired from parental experience has been demonstrated to occur through multiple mechanisms, such as germ-line and somatic transmission, fetal development, and maternal nurturing (Weaver et al., 2004; Champagne and Curley, 2009; Ho and Burggren, 2010; Meaney, 2010).

Epigenetic marks on chromatin can be inherited through maternal and paternal germ cells. Even if DNA methylation sites are generally erased during gonadal sex determination in embryogenesis, some of them survive and are integrated in the gametes persisting until the embryos become adult. In the present experimental model in which environmental manipulation was applied to mothers and not to fathers, epigenetic transgenerational effects occurred only through maternal germ-line, excluding any paternal influence.

Heritable epigenetic changes occur also through factors passed from mother to offspring via the placenta or milk, such as hormones, antibodies, antioxidants (Ho and Burggren, 2010). These factors have a significant impact on offspring phenotype, as indicated by studies showing how intrauterine milieu can affect offspring epigenome. For example, nutritional supplementation of folate in the maternal diet increases methyl donors in the circulation that cross the placenta and thus increases DNA methylation of many genes of offspring (Cooney, 2006; Cropley et al., 2006). Also in the present experimental model, the beneficial effects of pre-reproductive EE exposure may be epigenetically transmitted to the offspring before the birth *in utero* and after the birth by lactation.

Transgenerational epigenetic changes occur also through altered maternal nurturing. In fact, mother-infant interactions during the early life have a profound impact on offspring phenotype, as indicated by studies demonstrating that natural variations in the quality or quantity of maternal care leave long-lasting epigenetic marks on offspring molecular factors crucial for plasticity, such as BDNF and NMDA receptors (Weaver et al., 2004; Champagne and Curley, 2009). Furthermore, the adult offspring of mothers that exhibit increased levels of pup licking/grooming and arched back nursing show increased hippocampal glucocorticoid receptor expression and sensitivity and reduced stress reactions in comparison to adult animals reared by low licking/grooming mothers (Weaver, 2007). Also in the present experimental model, the pre-reproductive environmental manipulation could modify the features of the early mothering contributing thus to the accelerated acquisition of complex motor skills. Maternal behavior observation, cross-fostering, epigenetic analyses and their interaction may determine if the transgenerational inheritance is a product of prenatal factors or post-natal experience. Indeed, the relationship between behavior and epigenome is bilateral, behavior could result in epigenetic programming and epigenetic programming could affect behavior.

Overall, the present results demonstrating that pre-reproductive maternal environmental enrichment shapes the offspring phenotype indicate that females that experience a particular environment transgenerationally prepare their progeny to cope with that environment. Intriguingly, the transmission of maternal experience could have broad implications for progeny, improving their adaptive competencies and sculpting their behaviors (Petrosini et al., 2009).

## Author contributions

All authors designed research; Paola Caporali, Debora Cutuli, Daniela Laricchiuta, Francesca Foti and Laura Mancini performed behavioral evaluation; Francesca Gelfo, Francesco Angelucci, and Paola De Bartolo performed biochemical analyses; all authors analyzed data and discussed data; Laura Petrosini, Paola Caporali, Francesca Gelfo wrote the paper.

## Conflict of interest statement

The authors declare that the research was conducted in the absence of any commercial or financial relationships that could be construed as a potential conflict of interest.
