# Supplementary material for: Pre-reproductive maternal enrichment influences offspring developmental trajectories: motor behavior and neurotrophin expression
Source: Front Behav Neurosci. 2014 May 30;8:195. doi: 10.3389/fnbeh.2014.00195 (PMC4038762; doi:10.3389/fnbeh.2014.00195)
Supplement: Supplementary file 1 [file DataSheet1.DOC]

**Supplementary Table 1.** Statistical results of the analysis on development of quadrupedal locomotion.

| pnd | Quadrupedal locomotion |
| --- | --- |
| 3 | *z =* -1, *p* = 0.81 |
| 4 | *z* = 0.70, *p* = 0.63 |
| 5 | *z* = -1.53, *p* = 0.33 |
| 6 | *z* = 1.42, *p* = 0.63 |
| 7 | *z* = 0, *p* = 1 |
| 8 | *z* = -1.03, *p* = 0.63 |
| 9 | *z* = -1.77, *p* = 0.47 |
| 10 | *z* = 0.70, *p* = 0.66 |
| 11 | *z* = -0.35, *p* = 0.83 |
| 12 | *z* = 0.37, *p* = 0.81 |
| 13 | *z =* 0, *p* = 1 |
| 14 | *z =* 0, *p* = 1 |
