# Supplementary material for: Pre-reproductive maternal enrichment influences offspring developmental trajectories: motor behavior and neurotrophin expression
Source: Front Behav Neurosci. 2014 May 30;8:195. doi: 10.3389/fnbeh.2014.00195 (PMC4038762; doi:10.3389/fnbeh.2014.00195)
Supplement: Supplementary file 2 [file DataSheet2.DOC]

**Supplementary Table 2.** Statistical results of the analysis on swimming performance. Significant results are reported in bold.

| pnd | **Swimming performance** | |
| --- | --- | --- |
|  | Direction | Limb use |
| 1 | *z* = 1.53, *p* = 0.33 | *z* = -0.05, *p* = 0.97 |
| 2 | *z* = 0.50, *p* = 0.50 | *z* = -1.44, *p* = 0.20 |
| 3 | ***z* = 4.16, *p* < 0.001** | *z* = 2.19, *p* = 0.06 |
| 4 | *z* = 2.90, *p* = 0.01 | ***z* = 3.22, *p* < 0.01** |
| 5 | *z* = 0.16, *p* = 0.91 | *z* = 1.40, *p* = 0.47 |
| 6 | *z* = 1.62, *p* = 0.36 | *z* = 1.77, *p* = 0.47 |
| 7 | *z* = 2.50, *p* = 0.15 | *z* = -1, *p* = 0.81 |
| 8 | *z* = -0.73, *p* = 0.65 | *z* = 0, *p* = 1 |
| 9 | *z* = -0.73, *p* = 0.65 | *z* = 1, *p* = 0.81 |
| 10 | *z* = -2.07, *p* = 0.33 | *z* = 0, *p* = 1 |
| 11 | *z* = 0, *p* = 1 | *z* = 0, *p* = 1 |
| 12 | *z* = 1.10, *p* = 0.46 | *z* = 0, *p* = 1 |
| 13 | ***z* = 3.43, *p* < 0.01** | *z* = 0, *p* = 1 |
| 14 | *z* = 1.83, *p* = 0.22 | *z* = 0, *p* = 1 |
| 15 | *z* = 0.59, *p* = 0.81 | *z* = 0, *p* = 1 |
| 16 | *z* = 0, *p* = 1 | *z* = 0, *p* = 1 |
| 17 | *z* = 0, *p* = 1 | *z* = 1.77, *p* = 0.47 |
| 18 | *z* = 0, *p* = 1 | *z* = 2.07, *p* = 0.33 |
| 19 | *z* = 0, *p* = 1 | *z* = 2.34, *p* = 0.22 |
| 20 | *z* = 0, *p* = 1 | *z* = 2.56, *p* = 0.08 |
| 21 | *z* = 0, *p* = 1 | ***z* = 3.41, *p* < 0.01** |
| 22 | *z* = 0, *p* = 1 | *z* = 2.42, *p* = 0.05 |
| 23 | *z* = 0, *p* = 1 | *z* = 2.07, *p* = 0.33 |
| 24 | *z* = 0, *p* = 1 | *z* = 0, *p* = 1 |
| 25 | *z* = 0, *p* = 1 | *z* = 0, *p* = 1 |
